# An update on the antibiotic-based root canal irrigation solutions

**Published:** 2008-04-02

**Authors:** Zahed Mohammadi

**Affiliations:** 1 *Department of Endodontics, Dental School, Shahid Sadoughi University of Medical Sciences, Yazd, Iran*

**Keywords:** Antibiotics, Endodontic, Irrigation, Tetracycline

## Abstract

Antibiotics are a valuable addition to health practitioners for the management of bacterial infections. During endodontic treatment and when managing trauma to the teeth, antibiotics may be applied systemically or locally. Due to the potential risk of adverse effects of systemic applications, and the ineffectiveness of systemic prescribed antibiotics in necrotic or pulpless teeth and the periradicular tissues, the local application of antibiotics may be a more effective mode for delivering antibiotics to infected root canals. The purpose of this article is to review the history, rationale, and applications of antibiotics and antibiotic-containing irrigants in endodontics.

## Introduction

The role of microorganisms in the development and perpetuation of pulp and periapical diseases has clearly been demonstrated in animal models and human studies (1-3). Elimination of microorganisms from infected root canals is a complicated task. Numerous measures have been described to reduce the number of microorganisms in the root canal system (RCS), including the use of various instrumentation techniques, irrigation regimens and intra-canal medicaments. There is no definitive evidence in the literature that mechanical instrumentation alone results in a bacteria-free root canal system. Considering the complex anatomy of the root canal system (4), this is not surprising. On the contrary, there is *in vitro *and clinical evidence that mechanical instrumentation leaves significant portion of the root canal walls untouched (5) and that complete elimination of bacteria from the RCS by instrumentation alone is not achieved (6-9). Therefore, some additional methods, such as the use of chemical solutions, are required in order to disinfect the RCS and eliminate as many microorganisms as possible. Chemical treatment of the root canal system can be arbitrarily divided into irrigants, rinses, and inter-appointment medicaments. Several studies have been conducted on the use antibiotics as root canal irrigants. Hence, the purpose of this paper was to review the applications of antibiotics as root canal irrigants.

## History

Antibiotics were first discovered in 1928 but were not routinely used clinically until the early 1940s during the Second World War. Prior to this, most wartime deaths were due to bacterial infections of wounds, rather than from the wounds themselves. The use of antibiotics was popularized as a result of the rapid recovery of wounded military personnel and this popularity continued after the end of the war (10).

Antibiotics have been an extremely valuable addition to the armamentarium available to health practitioners for the management of bacterial infections. There is no doubt that they have often been used to save lives that would otherwise have been lost if antibiotics had not been available. For several decades antibiotics have been prescribed in different disciplines of medicine and dentistry (10). In endodontics and dental traumatology, antibiotics may be applied systemically (oral or parenteral) and locally (intra-dental). The first reported local use of an antibiotic in endodontics was in 1951 when Grossman (11) used a polyantibiotic paste known as PBSC (a mixture of penicillin, bacitracin, streptomycin, and caprylate sodium).

PBSC contained penicillin to target Gram-positive organisms, bacitracin for penicillin resistant strains, streptomycin for Gram-negative organisms, and caprylate sodium to target yeasts - these components were suspended in a silicone vehicle. Later, Nystatin replaced caprylate sodium as an antifungal agent in a similar medicament, known as PBSN (12).

## The rationale for local application of antibiotics

While systemic antibiotics appear to be clinically effective as an adjunct in certain surgical and nonsurgical endodontic cases, their administration is not without the potential risk of adverse systemic effects, such as allergic reactions, toxicity, various side effects and the development of resistant strains of microbes. In addition, systemic administration of antibiotics relies on patient compliance with the dosing regimens followed by absorption through the gastro-intestinal tract and then distribution via the circulatory system to bring the drug to the infected site. Hence, the infected area requires a normal blood supply which is no longer the case for teeth with a necrotic pulp, a pulpless and infected RCS or a root-filled tooth that become infected. Therefore, local application of antibiotics within the RCS may be a more effective mode for delivering the drug (13).

## Tetracyclines

Tetracyclines, including tetracycline-HCl, minocycline, demeclocycline and doxycycline, are a group of broad-spectrum antibiotics that are effective against a wide range of microorganisms (14). Tetracyclines are bacteriostatic in nature (14). This property may be advantageous because, in the absence of bacterial cell lysis, antigenic byproducts such as endotoxin are not released (15). Tetracyclines also have many unique properties other than their antimicrobial action, such as the inhibition of mammalian collagenases, which prevent tissue breakdown (16, 17), and the inhibition of clastic cells (17-19), which results in anti-resorptive activity (19). Inflammatory diseases such as periodontitis include an excess of tissue collagenases which may be blocked by tetracyclines, thus leading to enhanced formation of collagen and bone (15).

In periodontics, tetracyclines are used to remove the smear layer from instrumented root surfaces (*i.e.* dentine conditioning) and to remove surface contaminants such as bacterial endotoxins. The surface demineralization widens the orifices of the dentinal tubules and exposes the cementum collagen matrix which stimulates fibroblast attachment and growth (14). In endodontics, tetracyclines have been used to remove the smear layer from instrumented root canal walls (15, 20), for irrigation of retrograde cavities during periapical surgical procedures (21), and as an intracanal medicament (22). Barkhordar *et al. *(15) evaluated the effect of doxycycline-HCl on the smear layer of instrumented root canal walls. They showed that doxycycline-HCl eliminated smear layer in a concentration dependent manner with 100 mg/ml doxycycline being more effective than lower concentrations. In another investigation, Haznedaroglu and Ersev (20) used scanning electron microscopy (SEM) to assess the effect of tetracycline-HCl as an endodontic irrigant in removing the smear layer. They reported that tetracycline was as effective as citric acid in removing the smear layer. Barkhordar and Russell (21) evaluated the effect of doxycycline on the apical penetration of dye through the margins of retrograde fillings. The teeth with retrograde IRM or amalgam fillings placed subsequent to doxycycline irrigation had significantly less dye penetration than those that were not irrigated with doxycycline.

Carson *et al. *(23) used an agar diffusion test to compare the antimicrobial activities of 6% and 3% sodium hypochlorite (NaOCl) solutions, 2% and 0.12% chlorhexidine gluconate (CHX), and 0.01% and 0.005% doxycycline (Doxy) on four microorganisms associated with endodontic infections of teeth that had not been previously treated, namely *Peptostreptococcus micros*, *Prevotella intermedia*, *Streptococcus sanguis*, and *Lactobacillus acidophilus*. For the first three of these organisms, the general order of antimicrobial effectiveness was 0.01% Doxy >0.005% Doxy >6% NaOCl >3% NaOCl >2% CHX > 0.12% CHX. However, for *L. acidophilus*, the order of effectiveness was 6% NaOCl >3% NaOCl >2% CHX > 0.01% Doxy >0.005% Doxy >0.12% CHX. Pinheiro *et al.* (24) evaluated the antibiotic susceptibility of *Enterococcus faecalis *isolates from canals of root-filled teeth with periapical lesions. The antibiotics were benzylpenicillin, amoxicillin, amoxicillin with clavulanic acid, erythromycin, azithromycin, vancomycin, chloramphenicol, tetracycline, doxycycline, ciprofloxacin and moxifloxacin. The vast majority (85.7%) of the isolates were susceptible to tetracycline and doxycycline.

Chai *et al.* (25) investigated the antimicrobial efficacy of six groups of antibiotics (ampicillin, co-trimoxazole, erythromycin, oxytetracycline, vancomycin, and vancomycin followed by gentamicin) and calcium hydroxide against *Enterococcus faecalis *biofilm in a membrane filter model. They concluded that erythromycin, oxytetracycline and Ca (OH)2 were 100% effective in eliminating the *E. faecalis* biofilm, whereas ampicillin, co-trimoxazole, vancomycin, and vancomycin followed by gentamicin were ineffective. Based on the hypotheses that microorganisms can reach the apical area of recently replanted teeth from the oral cavity (or from contaminated root surfaces during the extra-oral time), and that tetracyclines can potentially inhibit this route of bacterial contamination, Cvek *et al.* (26) developed a protocol for the topical treatment of exposed roots with doxycycline before replantation. His aim was to eliminate the microorganisms from the root surface of an avulsed tooth via direct local application of the antibiotic in order to decrease the frequency and severity of the inflammatory response. They showed that topical doxycycline significantly increased the chances of successful pulp revascularization and decreased the number of microorganisms that could be isolated from the root canals. They also reported a decreased frequency of ankylosis, external replacement resorption and external inflammatory resorption. The beneficial effect of soaking a tooth in doxycycline has also been confirmed by Yanpiset and Trope (27).

Ritter *et al.* (28) investigated the effect of topical antibiotic treatment on pulp revascularization in replanted dogs’ teeth by using laser Doppler flowmetry (LDF), radiography and histology. After extraction, the teeth were kept dry for 5 minutes and either covered with minocycline, soaked in doxycycline, or soaked in saline and then they were replanted. Teeth in the positive control group were not extracted. Postoperative radiographs and LDF readings were obtained for 2 months after replantation. After sacrifice of the animals, the jaws were collected and processed for light microscopy. Pre- and post-replantation LDF readings and radiographs, and the histological findings were analyzed to assess revascularization. Pulp revascularization occurred in 91% of the teeth treated with minocycline, 73% of those soaked in doxycycline, and only 33% of the teeth soaked in saline.

Bryson *et al.* (29) evaluated the effect of minocycline on the healing of replanted dog teeth after extended dry times of 60 minutes. Their results indicated that the roots with and without minocycline treatment showed no significant differences in the remaining root mass or the percentage of favorably healed root surfaces. In addition, no benefit was found from the use of topically applied minocycline in the attenuation or prevention of external root resorption. The lack of significant differences is likely to have been a result of the extended dry period before replantation as most of the periodontal ligament cells would have died within this time period and therefore external replacement resorption is the typical result.

## Substantivity of tetracyclines

Tetracyclines readily attach to dentine and are subsequently released without losing their antibacterial activity (14). This property creates a reservoir of active antibacterial agent, which is then released from the dentine surface in a slow and sustained manner. In periodontics, several studies have been conducted on the antibacterial substantivity of tetracyclines. In an *in vivo *study, Stabholz *et al.* (30) compared the antibacterial substantivity of two concentrations of tetracycline HCl (50 mg/ml, 10 mg/ml) and 0.12% chlorhexidine. Their findings showed that both concentrations of tetracycline demonstrated residual antibacterial activity and the antibacterial substantivity of the three solutions in descending order was: 50 mg/ml tetracycline >10 mg/ml tetracycline > 0.12% CHX.

Abbott *et al.* demonstrated that tetracyclines form a strong reversible bond with the dental hard tissues and that they exhibit slow release over an extended period of time up to at least 12 weeks (31). Khademi *et al. *(32) compared the antibacterial substantivity of 2% CHX, 100 mg/ ml doxycycline-HCl and 2.6% NaOCl in bovine root dentine over five experimental periods of 0, 7, 14, 21 and 28 days *in vitro*. Their findings indicated that after 7 days, the NaOCl and doxycycline groups showed the lowest and the highest number of colony forming units (CFU), respectively. However, after the longer time periods, the CHX group showed the lowest number of CFU’s.

Mohammadi *et al.* (33) evaluated the antibacterial substantivity of three concentrations of doxycycline-HCl (100 mg/ml, 50 mg/ml, and 10 mg/ml) in bovine root dentine over five experimental periods of 0, 7, 14, 21 and 28 days. At 7 days, the 100mg/ml group and the 10mg/ml group showed the lowest and highest numbers of CFU’s, respectively. In each group, the numbers of CFU’s increased significantly over time.

## BioPure (MTAD)

Bio Pure (Dentsply, Tulsa Dental, Tulsa, OK, USA), otherwise known as MTAD (mixture of tetracycline, acid and detergent), is a relatively new root canal irrigant which was introduced by Torabinejad and Johnson (34) in 2003. This solution contains 3% doxycycline (at a concentration of 3%), citric acid (4.25%) and a detergent, Polysorbate 80 (0.5%) (34). Several studies have evaluated the effectiveness of MTAD for disinfection of root canals. Torabinejad *et al* have shown that MTAD is able to remove the smear layer (34) and is effective against *E. faecalis* (35-37).

Shabahang *et al. *(36) cleaned and shaped root canals of extracted human teeth and exposed them to human saliva. They then compared the antibacterial efficacy of a combination of 1.3% NaOCl as a root canal irrigant and MTAD as a final rinse with that of 5.25% NaOCl. Their findings showed that using MTAD in addition to 1.3% NaOCl was more effective at disinfecting root canals than using 5.25% NaOCl alone. However, Tay *et al.* (38) found that when MTAD was applied to 1.3% NaOCl-irrigated dentine, its antimicrobial substantivity was reduced. They attributed this to the oxidation of MTAD by NaOCl in a manner similar to the peroxidation of tetracycline by reactive oxygen species.

In another study, Shabahang and Torabinejad (37) compared the antibacterial effects of MTAD with those of NaOCl and EDTA by using standard *in vitro* microbiological techniques and they reported that MTAD was significantly more effective against *E. faecalis.* Kho and Baumgartner (39) compared the antimicrobial efficacy of 1.3% NaOCl /MTAD against *E faecalis* with that of the combined alternate use of 5.25% NaOCl and 15% EDTA for root canal irrigation. Bacterial samples taken early in the canal cleaning process revealed growth in none of the 20 samples irrigated with the 5.25% NaOCl/15% EDTA combination but 8 of the 20 samples irrigated with 1.3% NaOCl/ MTAD had bacterial growth. Further samples taken after additional canal enlargement revealed growth in none of 20 samples when 5.25% NaOCl/15% EDTA were used but there was still growth in 10 of the 20 samples when 1.3% NaOCl/MTAD was used. This investigation showed consistent disinfection of infected root canals when a combination of 5.25% NaOCl and 15% EDTA was used. However, the combination of 1.3% NaOCl/ MTAD left nearly 50% of the canals contaminated with *E. faecalis.*

Krause *et al.* (40) compared the antimicrobial effect of MTAD, two of its components (doxycycline and citric acid), and NaOCl against *E. faecalis* in two *in vitro* models using two different methods. In the tooth model, NaOCl and doxycycline were more effective than the control in killing *E. faecalis* at shallow bur depths into dentine, but at deeper depths, the NaOCl was superior. In the agar diffusion model, smaller inhibition zone of NaOCl to MTAD or doxycycline was observed. Ghoddusi *et al.* (41) evaluated the effect of MTAD as a final irrigant on bacterial penetration into the root canal system, and its interaction with two conventional root canal cements (AH-Plus and Rickert’s cement). They reported that it took longer for bacteria to penetrate the canals when either EDTA or MTAD was used for smear layer removal. Furthermore, the root canals filled with AH-Plus showed significantly longer duration of resistance to bacterial penetration than canals filled with Rickert’s cement.

Davis *et al.* (42) investigated the antimicrobial action of Dermacyn (Oculus Innovative Sciences, Petaluma, CA), MTAD, 2% CHX, and 5.25% NaOCl against *Enterococcus faecalis* using a zone of inhibition test. MTAD showed significantly larger zones of inhibition than 5.25% NaOCl, 2% CHX, and Dermacyn. Newberry *et al.* (43) determined the *in vitro* antimicrobial effect of MTAD as a final irrigant on eight strains of *E. faecalis* and they also measured the minimum inhibitory concentration (MIC) and the minimum lethal concentration (MLC) of MTAD. After irrigating with 1.3% NaOCl, the root canals and the external root surfaces were exposed to MTAD for five minutes. This treatment regimen was effective in completely eliminating growth of seven of the eight strains of *E. faecalis*. The MIC/MLC tests showed that MTAD inhibited growth of most strains of *E. faecalis *when diluted 1:8192 times and it killed most strains of *E. faecalis *when diluted 1:512 times.

Recently, Shabahang *et al. *(44) evaluated the effect of the addition of chlorhexidine to MTAD and the substitution of the doxycycline in MTAD with chlorhexidine to create a solution they named MCAD. They compared the effectiveness of these formulations at disinfecting extracted human teeth that had been infected with *E. faecalis. *None of the samples treated with standard MTAD or with the MTAD/chlorhexidine mixture showed the presence of residual bacteria. In contrast, 7 of the 10 samples treated with MCAD (doxycycline substituted by chlorhexidine) showed positive cultures of *E. faecalis.* These results clearly showed that, although the addition of chlorhexidine did not negatively impact on the efficacy of MTAD, the substitution of doxycycline with chlorhexidine significantly reduced the efficacy of the resultant solution.

## Substantivity of MTAD

As stated above, tetracyclines (including doxycycline) readily attach to dentine and are subsequently released without losing their antibacterial activity (14). The presence of doxycycline in MTAD suggests that MTAD may have some substantive antimicrobial action (14). In an *in vitro* study, Mohammadi and Yazdizadeh (45) evaluated the substantivity of NaOCl, CHX and MTAD using a bovine dentine tube model. Dentine chips were removed from the walls of root canals with sequential sterile low-speed round burs with increasing diameters of ISO sizes: 025, 027, 029, 031, and 033 at time intervals of 0, 7, 14, 21, and 28 days following irrigation with the test solution. In the first culture, the NaOCl group and the CHX group showed the lowest and highest number of CFU’s, respectively. In each group, the number of CFU’s increased significantly over time. The authors concluded that the substantivity of MTAD was significantly greater than CHX and NaOCl. These findings were also confirmed in a human dentine model (46). In another study, Mohammadi (47) assessed the substantivity of three concentrations (100%, 10%, and 1%) of MTAD using the bovine dentine tube model described above. In the first culture, the MTAD 100% group and the MTAD 1% group showed the lowest and highest number of CFU’s, respectively. In each group, the number of CFU’s increased significantly over time and it was concluded that the substantivity of 100% MTAD was significantly greater than the other two lower concentrations.

## Tetraclean

Tetraclean (Ogna Laboratori Farmaceutici, Muggiò (Mi), Italy), like MTAD, is a mixture of an antibiotic, an acid and a detergent. However, the concentration of the antibiotic, doxycycline (50 mg/ml), and the type of detergent (polypropylene glycol) differ from those of MTAD (48).

Giardino *et al. *(48) compared the surface tension of 17% EDTA, Cetrexidin, Smear Clear, 5.25% NaOCl, MTAD and Tetraclean. The NaOCl and EDTA had the highest surface tension, whereas Cetrexedin and Tetraclean had the lowest values. In another study, they compared the antimicrobial efficacy of 5.25% NaOCl, MTAD, and Tetraclean against an *E. faecalis* biofilm generated on cellulose nitrate membrane filters. Only the NaOCl could disaggregate and remove the biofilm at every time interval tested although treatment with Tetraclean caused a high degree of biofilm disaggregation at each time interval when compared with MTAD (49).

## Conclusions

**1.** The local application of antibiotics within the root canal system may be a more effective mode for delivering such drugs than systemic routes of administration.

**2.** Tetracyclines have been used to remove the smear layer from instrumented root canal walls, for irrigation of retrograde cavities during periapical surgical procedures, and as an intracanal medicament.

**3. **Substantivity of tetracyclines has been shown for up to at least 12 weeks.

**4.** BioPure (MTAD) is effective in removing the smear layer. However, the antimicrobial efficacy against *E faecalis *of 1.3% NaOCl/MTAD compared with that of the combined alternate use of 5.25% NaOCl and 15% EDTA is still controversial.

**5.** Substantivity of MTAD has been shown to last for up to 4 weeks. Furthermore, application of MTAD to 1.3% NaOCl-irrigated dentine may reduce its substantivity.

**6. **Tetraclean, is a mixture of an antibiotic (doxycycline), an acid, and a detergent (like MTAD), with a very low surface tension, and high degree of efficacy against bacterial biofilms.
